# ElectroKitty: A Python Tool for Modeling Electrochemical Data Including Non-Langmuir Adsorption

**DOI:** 10.1021/acselectrochem.4c00218

**Published:** 2025-04-15

**Authors:** Ožbej Vodeb, Pedro Farinazzo Bergamo Dias Martins, Dušan Strmčnik, Nejc Hodnik, Miran Gaberšček

**Affiliations:** † Department of Materials Chemistry, 68913 National Institute of Chemistry, Hajdrihova 19, 1000 Ljubljana, Slovenia; ‡ Jozef Stefan International Postgraduate School, Jamova cesta 39, 1000 Ljubljana, Slovenia; § University of Nova Gorica, Vipavska 13, 5000 Nova Gorica, Slovenia

**Keywords:** simulation, electrochemistry, voltammetry, data analysis

## Abstract

The use of simulation and various forms of data analysis is becoming more frequent in all areas of electrochemistry. To support this, we have created ElectroKitty, a Python package that can simulate complex reaction pathways. Since experimental data often exhibits non-idealities with adsorbed species, we have programmed ElectroKitty to incorporate such corrections. To demonstrate the versatility of our package we selected four common reaction pathways and validated it against an established simulator. We also demonstrate the Frumkin isotherm and how ElectroKitty can reproduce it. In addition, to demonstrate the more advanced features of our package, we used ElectroKitty to simulate OH adsorption on a Pt(111) surface using a straightforward model.

## Graphical abstract

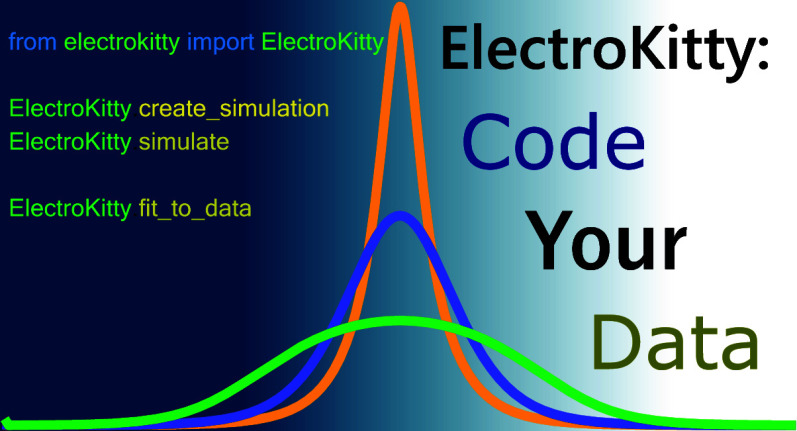

## Introduction

The basics of electrochemical data analysis include: finding current peaks, performing Tafel analysis, correcting uncompensated resistances, and sometimes even fitting models directly to measured data.^1^ Over the years, many different tools have been developed to process or even simulate data. Today, almost every potentiostat program has methods for processing data, with some having more advanced features than others.^2–4^ However, direct simulation of experiments is still not very common. A likely reason for this is that it is not straightforward to program a numerical algorithm that solves the governing equations, let alone fits the data. Unless an electrochemist has sufficient background in mathematics and computer science, fitting a model can be a daunting task.^5–7^

Numerous software packages have been developed over the years to facilitate simulation and data fitting. Some of the most well-known are DigiElch distributed by Gamry Instruments,^8,9^ MECSim developed at Monash University,^10,11^ and KISSA.^12^ All these simulators offer the possibility to compute electrochemical responses for different electrode geometries and to simulate different mechanistic networks. It is also worth mentioning that many different projects have emerged in recent years. Some of these projects are Soft Potato,^13,14^ FreeSim,^15^ Polarographica,^16^ and ecsim.^17^ Two other projects that should be mentioned here are RedoxPySolid,^18,19^ which was developed for the simulation of surface heterogeneity, and EchemFEM,^20,21^ a finite-element-based solver that can simulate the concentration, potential, and velocity profiles of a variety of geometries but has limitations when simulating different potential programs, adsorption, and surface-confined processes. For our comparison, we will exclude these two projects, as they do not fit well with the others since their use cases are very different compared to other software. In Table 1, we give a brief summary of the features offered by relevant projects and compare them with ElectroKitty, our own Python package.

**Table 1:** A brief comparison of different electrochemical simulation packages

**Name**	**Supported geometries**	**Data fitting**	**Supported mechanisms**	**Availability**
**MECSim**	Planar, spherical, RDE, cylindrical	Supported, further extended with BIOMEC^22,23^	Allows combining dissolved and adsorbed species	Free, open-source^10,11,22,23^
**DigiElch**	Planar, spherical, cylindrical, band, RDE	Supported	Allows combining dissolved and adsorbed species, includes adsorbate interaction	Proprietary, distributed by Gamry Instruments^9^
**KISSA**	Planar, hemispherical, hemicylindrical, band, RDE	Not-supported	Allows combining dissolved and adsorbed species, includes electrochemiluminescence modeling	Distributed by ProSense and BASi or by contacting the team^12^
**Soft Potato**	Planar, Band, Microdisk	Not-supported	Limited to predefined mechanisms	Free, open-source^13,14^
**Polarographica**	Can be complex	Supported	Limited to predefined mechanisms	Free, open-source^16^
**ecsim**	Planar, Disk, Spherical, Cylindrical, Hemispherical	Not-supported	Allows combining dissolved species	Free, open-source^17^
**FreeSim**	Planar, Spherical, with various boundary conditions	Not-supported	Limited to predefined mechanisms	Free, open-source^15^
**ElectroKitty**	Planar, RDE	Supported	Allows combining dissolved and adsorbed species, includes adsorbate interaction and thermodynamic dispersion	Free, open-source^24^

Based on the comparison, we can see that some packages can be quite versatile and can cover most use cases that an average electrochemist might have. However, almost all packages have something that they do not support or are closed sourced making them hard to integrate into larger projects. For this reason, we have developed ElectroKitty, which offers possibilities to simulate complex reaction networks, surface reactions with non-idealities, which are crucial for explaining surface oxidation and electrocatalysis, as well as offering ways to compare experiments with theory. To make ElectroKitty accessible, we have published it in Python, with all the source code and tutorials for the library available on GitHub, so it can be accessed or modified by anyone.^24^

To showcase the capabilities of ElectroKitty we demonstrated some of the most popular reaction pathways, before moving on to the effects of interactions between surface species and a “real-world” example of these effects. For such an example, we show the voltammogram of OH^–^ adsorption on a Pt(111) surface in 0.1 M HClO_4_ and attempt to fit the data with a simple model. Our aim is to show that we cannot “a priori” assume, even for a single-crystal electrode, Langmuir behavior and that models that incorporate surface interactions are necessary for a complete picture of the surface. Through such demonstrations, we wish to show that ElectroKitty can provide the community with a good starting point for the study of surface complexities. Moving forward, we plan to expand its capabilities, incorporating support for complex geometries and additional features to enhance its utility for the community.

## Capabilities of ElectroKitty

### Common Simulation Examples

To demonstrate the basic capabilities of ElectroKitty, we simulate four common reaction pathways and compare them with the results of MECSim. The pathways chosen are the E, EE, Ecat, and ECE reaction pathways (full mechanism show in Figure 1), with the ECE pathway showing the effect of uncompensated resistance. As can be seen in Figure 1, ElectroKitty can reproduce the currents provided by MECSim very well. The simulation details have been moved to the Supporting Information section 1, where the curious reader can find complete scripts for running the four examples. For more detailed instructions on how to use ElectroKitty, we refer the reader to ElectroKitty’s GitHub page.^24^

**Figure 1:**
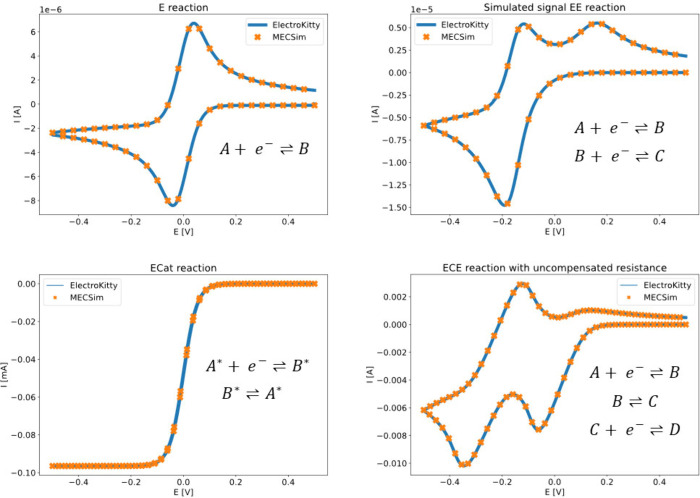
Comparison of simulation results for different reaction pathways between MECSim and ElectroKitty. The simulations can be reproduced using the scripts in Supporting Information section 1.

### Demonstrating the Effect of Adsorbate Interaction

In the literature, studies on various poly- or single-crystal surfaces have shown the importance of the interaction between adsorbed surface species in the observation of electrochemical reactions. Good examples are Hydrogen under-potential deposition and OH adsorption on Pt single-crystal electrodes,^25,26^ the oxidation and reduction of platinum, investigated by Conway.^27,28^ The authors observed effects of interaction between surface species and also consider these effects more generally.^29^ Other examples of electrochemical responses showing adsorbate interaction include the study of adsorption of oxygen on RuO_2_(110) single crystals,^30^ methanol oxidation on Rh and Ir electrodes,^31^ and recent studies of the Oxygen evolution reaction on Ir.^32,33^

These interactions can be modeled by adding an isotherm term to the rate equations.^29,34,35^ This is usually done by multiplying the rate law with an exponent of the coverage multiplied with the interaction parameter (g), see eq 1. In ElectroKitty we currently offer only the Langmuir and Frumkin isotherms, as their effects on electrochemical thermodynamics and dynamics are well documented. In principle, the Frumkin isotherm takes into account interactions between adsorbed species (e.g., repulsive), unlike the Langmuir isotherm, which does not assume such interactions. We note that there is a plethora of other isotherms with different rationales for their use that will be implemented into the package in the future.

1 $$\frac{d\theta_{i}}{dt}=-k_{f}\theta_{i}e^{-g_{i}\theta_{i}}+k_{b}\theta_{j}e^{-g_{j}\theta_{j}}$$

First, we demonstrate and validate our simulator by reproducing the interaction effect on a CV of a 1e^–^ adsorption reaction, as was done in Bard and Faulkner^34^ (see Figure 2a). We see that the interaction parameter has a large influence on the voltammogram, as the current peaks “stretch” or “shrink” to different degrees depending on the sign and value of the parameter. To further validate our simulator, we can try to reproduce the Frumkin isotherm, for a certain value of the interaction parameter. We demonstrate this in Figure 2b, where we have reproduced three isotherms, with the g value of 0 corresponding to a Langmuir isotherm, and the other two values to a Frumkin isotherm. The code for Figure 2 can be found in Supporting Information section 1.

**Figure 2:**
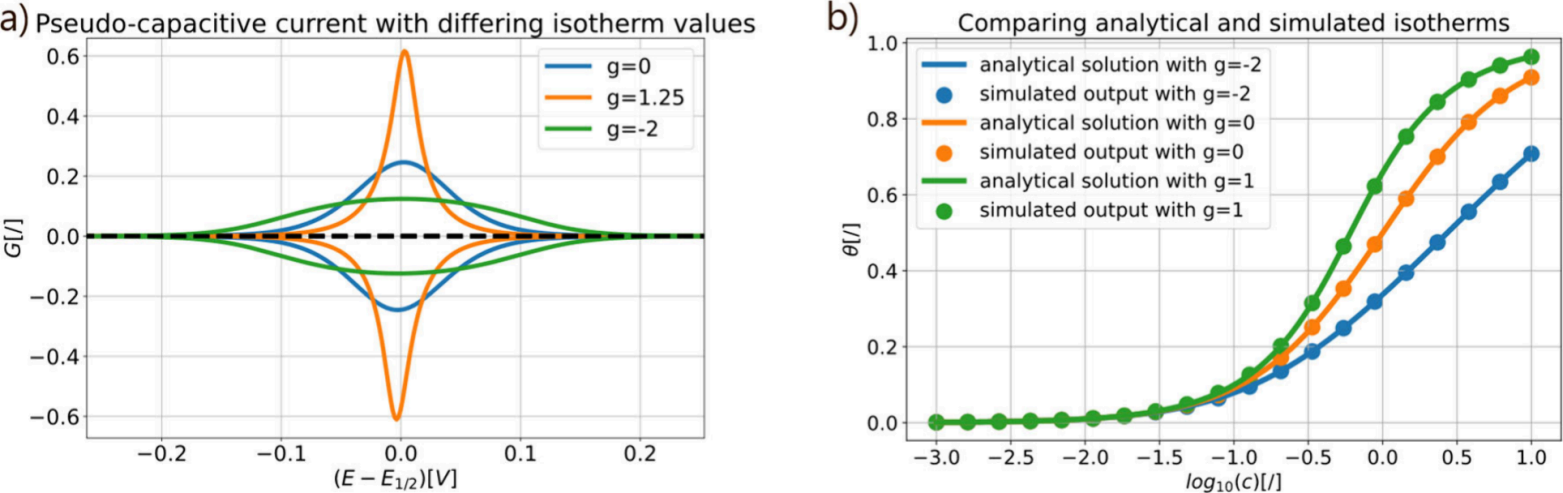
a) The effect of a Fumkin isotherm on the CV behavior of an adsorption process. Shown as a normalized current (G = i/nFAΓvf, with i as current, n the number of electrons, F the Faraday constant, A as electrode area, Γ as the maximum surface concentration, v as scan rate, and f = nF/*RT*, where T is temperature and R the gas constant) versus the potential. Following the convention of Bard,^34^ we see that a repulsive interaction leads to a broadening of the peak, while a positive value lengthens the peak. b) Frumkin isotherm modeled with ElectroKitty, without electron transfer, was adapted from a model in Schmickler et al.^35^ Here the coverage is plotted against the logarithm of the concentration. The equilibrium constant is assumed to be 1 in all cases, allowing it to be safely disregarded in the calculations. The scripts to recreate Figure 2 can be found in Supporting Information section 1, where all simulation parameters are listed.

### Demonstrating the Importance of Including Non-Ideal Behavior in Models

As an example of adsorbate interaction we chose to simulate OH adsorption on a Pt(111) single crystal surface. For experimental details, we refer the reader to Supporting Information section 2. Figure 3 shows experimental data that was used to fit an approximate model. As was discussed previously in the literature, the “butterfly region” of the voltammogram exhibits at least two electrochemical processes, neither of which is ideal.^25,26,36^ The first process shows a broadened peak, while the second shows a sharp peak, both peaks being a consequence of oxidation of two types of water, structured and unstructured. To fit this data, we employed the covariance matrix adaptation – evolution strategy (CMA-ES) algorithm, which ElectroKitty can use as its optimization algorithm.^37,38^ The model includes two electrochemical processes linked via a chemical process, that serves as an approximation of the adsorbates ordering. Such a model is far from perfect and serves only as a first approximation; for further discussion on the actual condition of the surface, we refer the reader to the relevant literature.^25,26,36,39–41^ The green curve in Figure 3 represents a fit where the interaction parameters were set to zero. Although ElectroKitty can approximate the experimental data to a certain extent, the fit is not entirely accurate. With the orange curve, we allow ElectroKitty to optimize non-ideal parameters as well, resulting in a significantly improved match with the data. This is also confirmed by the fit scores of both cases, with the non-ideal case having a fit score about 4.5 times better than the ideal case. In the ideal case, the relative root mean squared error (RRMSE) is 0.37, while in the non-ideal case, it is 0.08, turning the fit from unacceptable to acceptable.^42^

**Figure 3:**
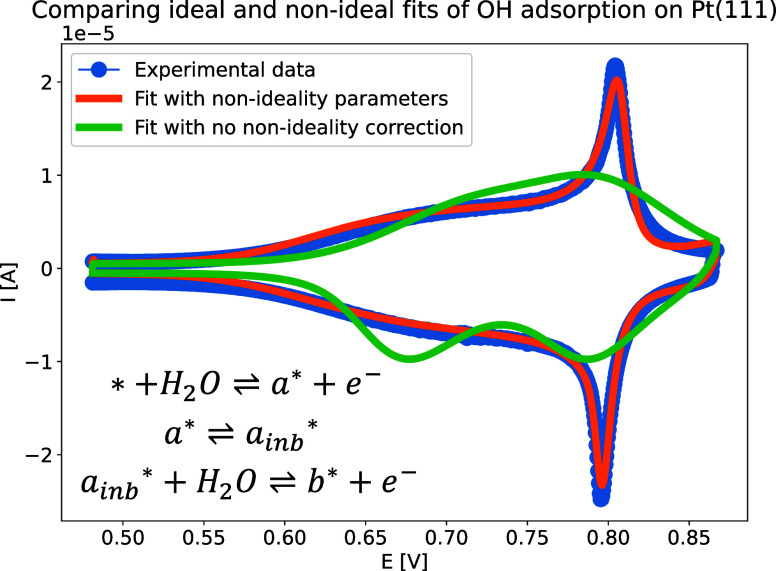
Experimental data of OH adsorption on a Pt(111) surface in 0.1 M perchloric acid (blue curve). The CV is recorded between 0.48 and 0.87 V vs. RHE, with a scan rate of 0.05 Vs^–1^. The mechanism of the simulation is found in the figure. The orange curve represents parameter optimization that includes non-ideality parameters, while in the case of the green curve, those are set to zero, representing adsorption without adsorbate interaction.

The data analysis can also be extended with Bayesian inference, which is also included with ElectroKitty, based on a procedure found in.^43^ We can estimate our parameter uncertainties using Bayesian inference by sampling the distribution of our fitting parameters. The results can be found in the Supporting Information section 3 (Figure S1) shown by a pairwise plot for all of our parameters and the standard deviation of experimental noise. These sorts of plots can also determine whether our model is suitable, as a model showing high scattering of parameters is likely wrong (Figure S1a), as is the case in the model without any non-ideality corrections. An argument would again be that we are overfitting our data by adding additional parameters to our model; however, as can be seen from the inference plot (Figure S1b), that is likely not the case as all parameters show a moderate amount of scattering. We refer the reader to Supporting Information section 3 for further discussion on the result.

## Conclusions and outlook

With this technical note, we have introduced ElectroKitty, a Python package capable of simulating various mechanisms and then fitting the mechanism to real data. We have demonstrated the basic functionalities of ElectroKitty by simulating four reaction pathways that are common in electrochemistry. We have also shown how to simulate OH adsorption on a Pt(111) surface. We have fitted this simple model to our data and extended the fit with Bayesian Inference. Although ElectroKitty can be a powerful tool, it also has some drawbacks. For example, it requires the user to be able to program in Python. Although some other packages still offer certain advanced functionalities, we believe that ElectroKitty serves as a strong starting toolbox for tackling complex surface reactions. In conclusion, we welcome anyone who would like to contribute to the project in any way. Contributions can be made by submitting a pull request on our GitHub or contacting the corresponding author.^24^ There is still a lot to be done. Let this be a project made by the electrochemical community for the electrochemical community.

## Supplementary Material



## Notes

Experimental details, codes, and further validations (PDF)
