# Supplementary material for: ElectroKitty: A Python Tool for Modeling Electrochemical Data Including Non-Langmuir Adsorption
Source: ACS Electrochem. 2025 Apr 15;1(8):1540–4. doi: 10.1021/acselectrochem.4c00218 (PMC12337095; doi:10.1021/acselectrochem.4c00218)
Supplement: Supplementary file 1 [file ec4c00218_si_001.pdf]

Supporting Information for technical note: ElectroKitty: A Python tool for modeling electrochemical data including non-Langmuir adsorption

Ožbej Vodeb[1,2]*, Pedro Farinazzo Bergamo Dias Martins[1], Dušan Strmčnik[1], Nejc Hodnik[1,2,3], Miran Gaberščček[1]

Department of Materials Chemistry, National Institute of Chemistry, Hajdrihova 19, 1000 Ljubljana, Slovenia[1]

Jozef Stefan International Postgraduate School, Jamova cesta 39, 1000 Ljubljana, Slovenia[2]

University of Nova Gorica, Vipavska 13, 5000 Nova Gorica, Slovenia[3]

Corresponding author:

* Department of Materials Chemistry, National Institute of Chemistry, Hajdrihova 19, 1000 Ljubljana, Slovenia, Email: Ozbej.Vodeb@ki.si

Contents

1. Codes to simulate the reactions shown in the main text S2
   - The E reaction: S2
   - The EE reaction: S2
   - The Ecat reaction: S3
   - The ECE reaction: S4
   - The code to recreate the Figure 2a of the main text. S5
   - The code to recreate Figure 2b S6
2. Experimental details S8
3. Bayesian Inference results for both models S8
4. Verifying the simulator S10
5. References S13

1. Codes to simulate the reactions shown in the main text

Below the codes to simulate the four reactions in Figure 1 of the main text are given.

The E reaction:

```
import numpy as np
import matplotlib.pyplot as plt
from electrokitty import ElectroKitty

nx=20

mechanism="E(1): a=b"

kin_const=[
    [0.5, 10**-2, 0],
 ]

viscosity=10**-5 #m^2/s
rot_freq=0 #Hz

cell_c=[298, 1000, 1*10**-5, 10**-5] # K, Ohm , F/m^2, m^2

si=[0.001/36, nx, viscosity, rot_freq]

spec_info=[[],
           [1,0]]

iso=[]

Ei=0.5 #Initial potential, also the potential to wich the CV returns [V]
Ef=-0.5 #Final potential, we cycle to this potential [V]
v=0.01   #scan speed [V/s]
freq=9 #In case of ACV this is the frequency of a sine wave [Hz]
amp=0.0  #In case of ACV this is the amplitude of a sine wave [V]
nt=1000 #number of time points

problem=ElectroKitty(mechanism)
problem.V_potencial(Ei, Ef, v, amp, freq, nt)
problem.create_simulation(kin_const, cell_c, D, iso, si, spec_info)
problem.simulate()
problem.Plot_simulation()
```

The EE reaction:

```
import numpy as np
import matplotlib.pyplot as plt
from electrokitty import ElectroKitty
```

```
nx=20

mechanism="E(1): a=b \n E(1):b=c"

kin_const=[
    [0.5, 10**-6, 0],
    [0.5, 10**-2, -0.15]
 ]

D=3*[10**-9]

viscosity=10**-5 #m^2/s
rot_freq=0 #Hz

cell_c=[298, 10, 0*10**-5, 10**-5] # K, Ohm , F/m^2, m^2

si=[0.001/36, nx, viscosity, rot_freq]

theta=1

spec_info=[[],
           [1,0,0]]

iso=[]

spe=[[1,1],[1,0]]

Ei=0.5 #Initial potential, also the potential to wich the CV returns [V]
Ef=-0.5 #Final potential, we cycle to this potential [V]
v=0.01   #scan speed [V/s]
freq=9 #In case of ACV this is the frequency of a sine wave [Hz]
amp=0.0  #In case of ACV this is the amplitude of a sine wave [V]
nt=1000 #number of time points

problem=ElectroKitty(mechanism)
problem.V_potencial(Ei, Ef, v, amp, freq, nt)

problem.create_simulation(kin_const, cell_c, D, iso, si, spec_info)

problem.simulate()
problem.Plot_simulation(label = "ElectroKitty", x_label = "E [V]", Title = "EE reaction")
```

The Ecat reaction:

```
import numpy as np
import matplotlib.pyplot as plt
from electrokitty import ElectroKitty
```

```
nx=20

mechanism="E(1):c*=d* \n C:d*=c*"

kin_const=[
    [0.5, 10**4, 0],
    [10**1,10**-20]
 ]

D=[]

viscosity=10**-5 #m^2/s
rot_freq=0 #Hz

cell_c=[298, 10, 1*10**-6, 10**-5] # K, Ohm , F/m^2, m^2

si=[0.001/36, nx, viscosity, rot_freq]

spec_info=[[10**-5,0],
           []]

iso=[0,0]
spe=[[1,1],[1,0]]

Ei=0.5 #Initial potential, also the potential to wich the CV returns [V]
Ef=-0.5 #Final potential, we cycle to this potential [V]
v=0.01   #scan speed [V/s]
freq=9 #In case of ACV this is the frequency of a sine wave [Hz]
amp=0.0  #In case of ACV this is the amplitude of a sine wave [V]
nt=1000 #number of time points

problem=ElectroKitty(mechanism)
problem.V_potencial(Ei, Ef, v, amp, freq, nt)
problem.create_simulation(kin_const, cell_c, D, iso, si, spec_info)
problem.simulate()
problem.Plot_simulation()
```

The ECE reaction:

```
import numpy as np
import matplotlib.pyplot as plt
from electrokitty import ElectroKitty

nx=20

mechanism="E(1):a=b \n C:b=c \n E(1): c=d"

kin_const=[
    [0.5, 10**-2, 0.],
```

```
    [10**1,10**-4],
    [0.5, 10**-2, -0.2],
  ]

D=4*[10**-9]

viscosity=10**-5 #m^2/s
rot_freq=0 #Hz

cell_c=[298, 0, 0*10**-6, 10**-5] # K, Ohm , F/m^2, m^2

si=[0.0001/36, nx, viscosity, rot_freq]

spec_info=[[],
           [1,0,0,0]]

iso=[]

spe=[[1,1],[1,0]]

Ei=0.5 #Initial potential, also the potential to wich the CV returns [V]
Ef=-0.5 #Final potential, we cycle to this potential [V]
v=0.01   #scan speed [V/s]
freq=9 #In case of ACV this is the frequency of a sine wave [Hz]
amp=0.0  #In case of ACV this is the amplitude of a sine wave [V]
nt=1000 #number of time points

problem=ElectroKitty(mechanism)
problem.V_potencial(Ei, Ef, v, amp, freq, nt)
problem.create_simulation(kin_const, cell_c, D, iso, si, spec_info)
problem.simulate()
problem.Plot_simulation()
```

The code to recreate the Figure 2a of the main text.

```
import numpy as np
import matplotlib.pyplot as plt
from electrokitty import ElectroKitty

F=96485 #As/mol
R=8.314 #J/mol/K
T=298 #K

mechanism="E(1): a+*=a*"

kin_const=[
    [0.5, 1000, 0],
 ]
```

```
D=1*[10**-8]

viscosity=10**-5 #m^2/s
rot_freq=0 #Hz

cell_c=[T, 0, 0*10**-6, 10**-4] # K, Ohm , F/m^2, m^2

si=[0.001/36, 20, viscosity, rot_freq]

spec_info=[[10**-5,0],
           [1]]

Ei=0.3 #Initial potential, also the potential to wich the CV returns [V]
Ef=-0.3 #Final potential, we cycle to this potential [V]
v=0.05   #scan speed [V/s]
freq=9 #In case of ACV this is the frequency of a sine wave [Hz]
amp=0.0  #In case of ACV this is the amplitude of a sine wave [V]
nt=1000 #number of time points

isos = [0, 1, -2]

for param in isos:
    iso=[param, param]
    problem=ElectroKitty(mechanism)
    problem.V_potential(Ei, Ef, v, amp, freq, nt)
    problem.create_simulation(kin_const, cell_c, D, iso, si, spec_info)
    problem.simulate()

    plt.plot(problem.E_generated, problem.current/(F**2/R/T*v*cell_c[-
1]*spec_info[0][0]), linewidth = 4.5, label=f"g={iso[0]}")
    plt.xlabel("$(E-E_{1/2}) [V]$")
    plt.ylabel("$G [/]$")
plt.grid()
plt.legend()
```

The code to recreate Figure 2b

Please note that the two books cited use different sign conventions when describing repulsive or attractive Frumkin effect. ElectroKitty by default uses the convention that a negative constant corresponds to a repulsive interaction on the surface, as such the model given in Interfacial Electrochemistry is modified slightly to conform to our convention.

```
import numpy as np
import matplotlib.pyplot as plt
from electrokitty import ElectroKitty
import scipy.optimize as sciop

def eq(x, c, g):
    f=x/(1-x)*np.exp(-g*x)-c
```

```
    return f

def calc_coverage(cmax, g):
    F=96485 #As/mol
    R=8.314 #J/mol/K
    T=298 #K

    mechanism="C: a+*=a*"

    kin_const=[
        [1,1],
     ]

    D=1*[10**-8]

    viscosity=10**-5 #m^2/s
    rot_freq=0 #Hz

    cell_c=[T, 0, 0*10**-6, 10**-4] # K, Ohm , F/m^2, m^2
    si=[0.001/36, 20, viscosity, rot_freq]

    spec_info=[[10**-5,0],
               [float(cmax)]]

    iso=[0,float(g)]

    problem=ElectroKitty(mechanism)
    problem.C_potential(0, 1000, 1000)
    problem.create_simulation(kin_const, cell_c, D, iso, si, spec_info)
    problem.simulate()
    theta = problem.surface_profile
    return theta[-1,1]/10**-5

cs = np.logspace(-3,1,100)

gs = [-2,0,1]

for g in gs:
    thetam = []
    for c in cs:
        sol = sciop.root(eq, [0.1], args=(c,g), tol=10**-12)
        thetam.append(sol.x[0])

    plt.plot(np.log10(cs), thetam, linewidth = 4.5, label = f"analytical
solution with g={g}")

    cs1 = np.logspace(-3,1,20)
    theta_sim=[]
    for c in cs1:
```

```
        theta_sim.append(calc_coverage(c, g))

    plt.scatter(np.log10(cs1), theta_sim, label = f"simulated output with
g={g}", linewidths = 7.5)

plt.grid()
plt.legend()
plt.xlabel(r"$log_{10}(c) [/]$")
plt.ylabel(r"$\theta [/]$")
plt.title("Comparing analytical and simulated isotherms")
```

2. Experimental details

Electrochemical measurements were conducted in 0.1 M perchloric acid solution prepared using ultra-pure deionized water (R ≥ 18.2 MΩ cm, Milli-Q system) and ultra-high purity $HClO_4$ (OmniTrace Ultra, EMD). The electrolyte, maintained at ~293 K, was purged with ultra-high purity argon gas (99.9999%, Airgas), and the potential sweep rate was set to 50 mV s^{-1}. A platinum wire (99.997%, Alfa Aesar) served as the counter electrode, and a silver/silver chloride electrode saturated with potassium chloride (BASi) was used as the reference. All potentials are reported against the reversible hydrogen electrode (RHE), which was calibrated separately under identical electrochemical conditions, except the electrolyte was purged with ultra-high purity hydrogen gas (99.9999%, Airgas). The single-crystalline platinum surface, Pt(111), was prepared as described previously.[1] Briefly, a Pt(111) disc (6 mm diameter, 4 mm height, MaTecK GmbH) was annealed at ~1,473 K for 7 min in a controlled 3% H_2/97% Ar atmosphere (~1 atm, Airgas) using a radio frequency induction system (EASYHeat, Ambrell) and cooled slowly (~7 min) under the same conditions. To protect the surface from contamination, a water droplet was placed on the Pt(111) surface before exposure to the laboratory atmosphere. The disc was then carefully mounted in a hanging meniscus configuration to prevent air exposure and attached to a rotating shaft connected to a rotator (MSR, Pine Research). Before electrochemical measurements, the Pt(111) surface was immersed in the electrolyte under a controlled potential of 0.05 V.

3. Bayesian Inference results for both models

As was demonstrated in the article including parameters for surface non-ideality is necessary in the case of OH^- adsorption on a Pt(111) surface. The parameters correct the model for any potential interaction of the surface-confined species. In this case, the first reaction exhibits a repulsive interaction, while the second is an attractive one. The parameters used here are purely empiric and do not hold much significant physical meaning, besides being a correction to the model. To demonstrate the necessity of including such corrections we compare the results of using Bayesian Inference to sample the parameter distributions of our two models. The constants fitted were in both cases all kinetic constants, the double-layer capacitance, and maximum surface concentration, in the non-ideal case four more constants were added to correct the current response.

As was noted in the main article the first obstacle for the ideal surface model is the RRMSE value, as it is too high for it to be deemed an acceptable fit. The second obstacle appears when examining the pairwise plot in Figure S1a. We can see that the k_{f2} and k_{b2}, the constants for the chemical reaction, both exhibit large variance. The same is true for $k_{0,1}$ and α_1 values, the double layer capacitance and maximum surface concentration. Thus, leading us to doubt the validity of such a model. To put it in

another way: While there exists a maximum-likelihood value for each parameter, the fact that so many different values were deemed “good enough” makes our model suspicious, as the range of acceptability should be as narrow as possible.

On the other hand, the model that assumes a non-ideal surface exhibits low variance of its parameters. Thus, we can have high confidence that our fit and thus our model is not entirely wrong. Many parameters exhibit strong correlations, showing that the underlying distribution of the parameters is indeed complex and that fitting all of the parameters at the same time is a difficult process. These correlations do not however implicate that a physical law is the cause of such behavior. Interestingly, the standard deviation of the noise is also about an order of magnitude lower, which is likely the consequence of the model being able to simulate both the sharp and broad peak, thus not assigning points it cannot “reach” as noise.

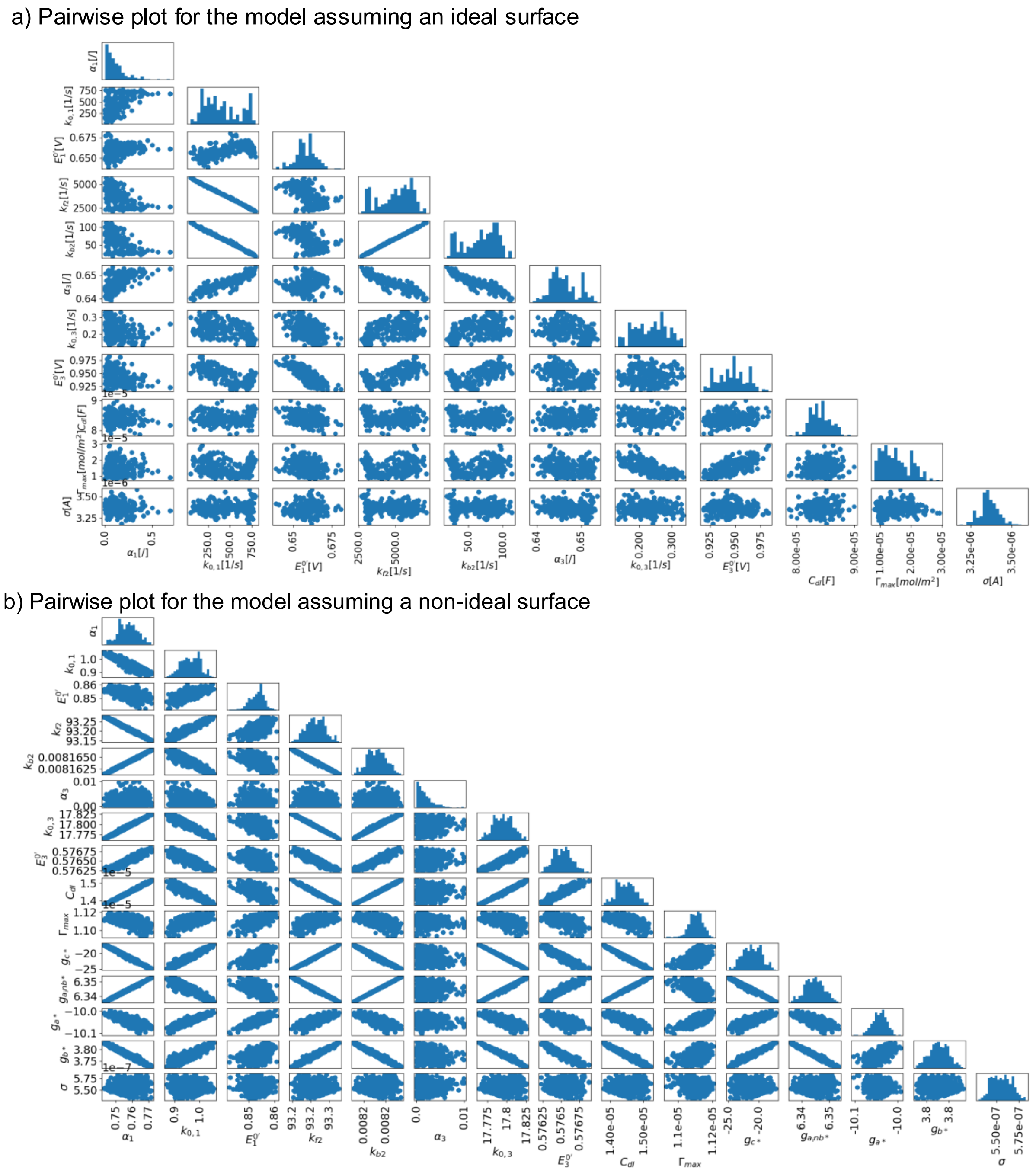

Figure S1: Pairwise plots after burn-in for both models presented in the main article. a) The plot for the model assuming an ideal surface. The g parameters were kept at 0 during fitting and inference. b) The plot for the model that assumes a non-ideal surface. The 4 additional parameters are the g parameters that correct for surface non-idealities.

4. Verifying the simulator

A good simulator is only as good as the accuracy with which it can simulate electrochemical data. Here we want to show that the ElectroKitty simulator fulfills the necessary criteria to use it as a simulation tool. To achieve this, we first compare the simulation results with some analytical results from Bard[2]. We start with the simulation of a simple 1-electron process at a planar electrode confined by diffusion. In the reversible limit, the peak currents should follow the Randles-Ševčik equation. The dimensionless current should be -0.4463 and should be found at an overvoltage of ±28.5 mV. As can be seen from Figure S2, the simulator approximates the analytical values in a reasonable way. The dimensionless

current has an error of 0.09 %, while the potential is off by about 2.4 %. This is to be expected as determining the correct potential value is much more difficult than estimating the current, as mentioned earlier.[3]

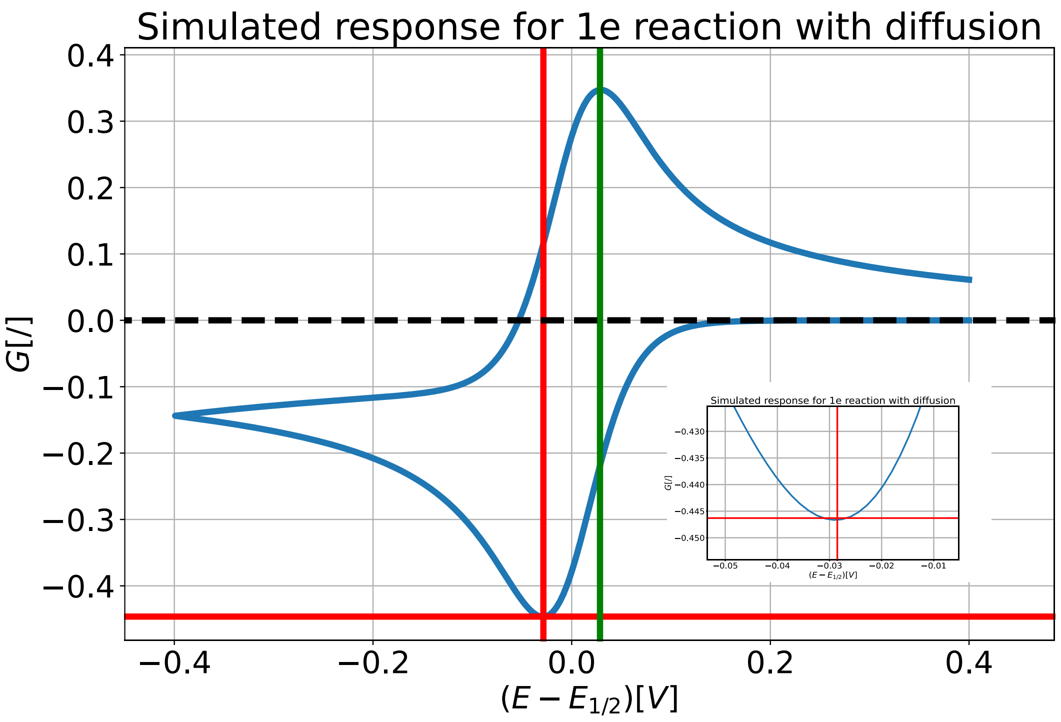

Figure S2: Diffusion limited current with red and green lines showing the overpotential of 28.5 mV. The horizontal line shows the predicted current maxima. The inset is a closeup at the maxima.

Next, we consider the pseudo-capacitive case. The normalized peak currents should be equal to 0.25 for an overpotential of 0 V (Figure S3). From the inserted figure we can see that the simulator does not quite reach the expected value. The simulation error is therefore given as 1.6 %, with 1000 simulation points. It should be noted that in both cases discussed so far, the error can be minimized by increasing the number of points in time. The validation of the Frumkin isotherm is shown in Figure 2 of the main article.

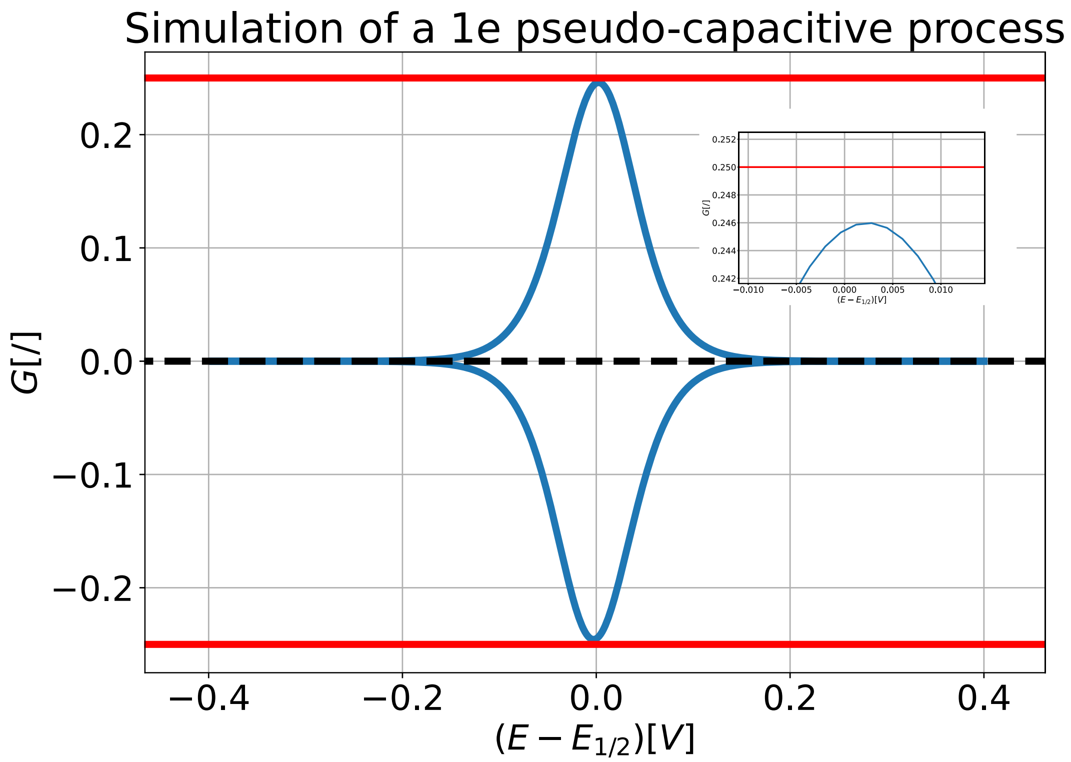

Figure S3: Simulation of a reversible pseudo-capacitive response. The inset better demonstrates the deviation from the analytical solution.

Furthermore, we validate that the simulator can accurately predict the behavior of an RDE. This can be easily achieved by comparing the limiting current determined by the simulation with the Levich equation. Figure S4 shows the simulated results for different rotation frequencies, the limiting current given by Levich for each frequency is shown as a dashed black line. The average error for all tests is 0.15 %, with the error increasing with increasing rotational frequency.

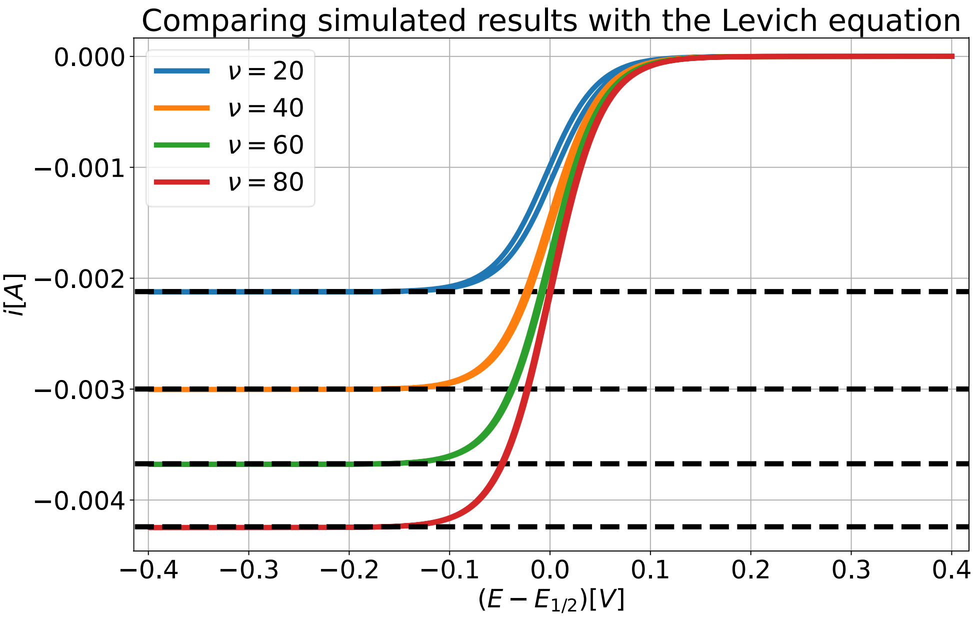

Figure S4: Comparing the limiting current of an RDE simulation for different rotational frequencies with the Levich equation.

5. References

(1) Strmcnik, D. S.; Tripkovic, D. V.; van der Vliet, D.; Chang, K.-C.; Komanicky, V.; You, H.; Karapetrov, G.; Greeley, J. P.; Stamenkovic, V. R.; Marković, N. M. Unique Activity of Platinum Adislands in the CO Electrooxidation Reaction. *J Am Chem Soc* **2008**, *130* (46), 15332–15339. https://doi.org/10.1021/ja8032185.

(2) Bard, A. J. ; F. L. R.; White S. Henry. *Electrochemical Methods: Fundamentals and Applications*, 3rd Edition.; John Wiley $ Sons, inc.: Austin, Texas, 2022.

(3) Britz, Dieter; Strutwolf, J. *Digital Cyclic Voltammetry*, Fourth Edi.; Scholz, F., Ed.; Springer US: Aarhus, Dennmark, 2016.
